# Observed Manipulation Enhances Left Fronto-Parietal Activations in the Processing of Unfamiliar Tools

**DOI:** 10.1371/journal.pone.0099401

**Published:** 2014-06-09

**Authors:** Norma Naima Rüther, Marco Tettamanti, Stefano F. Cappa, Christian Bellebaum

**Affiliations:** 1 Institute of Cognitive Neuroscience, Dept. of Neuropsychology, Ruhr University Bochum, Bochum, Germany; 2 International Graduate School of Neuroscience, Ruhr University Bochum, Bochum, Germany; 3 Division Neuroscience, San Raffaele Scientific Institute, Milano, Italy; 4 Department of Nuclear Medicine, San Raffaele Scientific Institute, Milano, Italy; 5 Faculty of Psychology, Vita-Salute San Raffaele University, Milano, Italy; 6 Institute of Experimental Psychology, Heinrich Heine University Düsseldorf, Düsseldorf, Germany; Weill Cornell Medical College, United States of America

## Abstract

Tools represent a special class of objects, as functional details of tools can afford certain actions. In addition, information gained via prior experience with tools can be accessed on a semantic level, providing a basis for meaningful object interactions. Conceptual representations of tools also encompass knowledge about tool manipulation which can be acquired via direct (active manipulation) or indirect (observation of others manipulating objects) motor experience. The present study aimed to explore the impact of observation of manipulation on the neural processing of previously unfamiliar, manipulable objects. Brain activity was assessed by means of functional magnetic resonance imaging while participants accomplished a visual matching task involving pictures of the novel objects before and after they received object-related training. Three training session in which subjects observed an experimenter manipulating one set of objects and visually explored another set of objects were used to make subjects familiar with the tools and to allow the formation of new tool representations. A control object set was not part of the training. Training-related brain activation increases were found for observed manipulation objects compared to not trained objects in a left-hemispheric network consisting of inferior frontal gyrus (iFG) pars opercularis and triangularis and supramarginal/angular gyrus. This illustrates that direct manipulation experience is not required to elicit tool-associated activation changes in the action system. While the iFG activation might indicate a close relationship between the areas involved in tool representation and those involved in observational knowledge acquisition, the parietal activation is discussed in terms of non-semantic effects of object affordances and hand-tool spatial relationships.

## Introduction

To interact meaningfully with objects in the environment, individuals must be able to identify interaction sites that are part of the objects' appearances, an object-characteristic that is often related to the influential concept of “affordances” [1]. This concept states that the environment contains inherent cues offering possibilities to act upon, hence influencing perceptual processing. Tools are a special class of objects, because they have intrinsic manipulability. On the other hand, however, the exact function of a tool and the way in which it is manipulated must be learned. The neural organization of tool concepts in semantic memory is a central topic in cognitive neuroscience which is still unresolved. Many neuroimaging studies have shown activations in a fronto-parietal network during the processing of tool stimuli, suggesting that regions involved in tool-related actions are activated when pictures of tools are seen or tool sounds are heard. It appears that this activation reflects the automatic recruitment of underlying neural motor patterns related to hand movements and grasping as well as the access to object-associated goals, which are integrated into tool concepts (e.g., [2]–[6]; for review, see [7]). In support of this view, Grezes and Decety (2002) showed that the processing of tool-stimuli leads to the activation of left inferior parietal lobule (iPL) and left inferior frontal gyrus (iFG; BA45), irrespective of a specific task [8].

Behavioral studies in healthy human subjects [2], [9], [10] provide only indirect evidence for an automatic motor cortex activation during the observation of tool stimuli, as shown by motor facilitation effects through tool picture presentations that can be attributable to the objects' inherent affordance cues. Studies using electroencephalography (EEG) show an early effect within 140–270 milliseconds (ms) that was interpreted as automatic affordance extraction [11], [12].

Important insights on the neural underpinnings of tool use and tool representations can be gained by studying patients suffering from apraxia (for review see [13]). The use of tools and objects is affected in a large proportion of apraxic patients, and follows from damage to the left frontal and temporo-parietal cortex [14]. Some apraxic patients may misuse common tools, for example trying to cut a piece of paper with closed scissors [15]–[17]. These symptoms have been related by some researchers [15], [16] to an impairment of stored conceptual knowledge (“action semantics”). Knowledge about object function has been suggested to contribute, together with general action knowledge and knowledge about action sequences, to a conceptual praxis system which, in turn, builds so-called action semantics along with a praxis production system [18]–[20]. The “sensory-motor theory” [21], [22] proposes that object concepts are stored in the brain regions that were active during knowledge acquisition, thus ascribing a central role to individual object-related experience [21], [23], [24]. In accordance with this notion, models of praxis have postulated that prior experience leads to a “processing advantage” for each new experience, based on action semantics in memory, which entail not only concrete information on object function but possibly also on manipulation [19]. The recruitment of fronto-parietal brain regions in the processing of tool stimuli should thus not only reflect affordance related processes, but can also be related to previous sensory-motor experience with the objects, reflecting a reactivation of regions involved in acquisition of knowledge about the object. Several studies used training procedures with novel objects to control for individual object experience, aiming to elucidate the impact of modality-specific experience on conceptual representations of those objects [25]–[29]. Indeed, it was shown that after short periods of training, fronto-parietal brain regions were recruited during visual processing of the novel objects in an experience-dependent manner. Only when subjects learned to manipulate the objects, but not when they visually explored them, stronger post training activations were seen in the premotor and the posterior parietal cortex [27], [29]. Experience effects are not specific for tools. A recent study in which participants were trained in either tying or naming knots showed recruitment of bilateral intraparietal sulcus (IPS) post training only when knots had been tied previously. In contrast, the left posterior IPS was active for knots that were learned to be named, showing that learning object-related information by linguistic or manipulation training leads to a recruitment of the parietal cortex independently of training experience [25].

Representations of tools, including information of associated action goals and manipulation sites, can be induced not only by active manipulation experience or, at least in part, by linguistic information, but also through indirect object experience, via observation of object manipulation, an important mechanism during the evolution of tool use behavior [30]. Models of praxis processing suggest that knowledge about tool use and function can be acquired via different routes [18], [19], leaving open the issue whether direct object experience is necessary for tool representations to emerge. As outlined above, tools represent a class of objects that is mainly defined by their intrinsic properties that afford to interact and are associated with goal-relevant, conceptual information [31]. Changes in tool-associated brain activation elicited by active manipulation experience, as those described above, can be ascribed to a change in object affordances. Recently, it was shown that expectations about tool use behaviors can be modulated by an interaction of biomechanical affordance cues and experience-dependent probabilistic priors of observed goals coupled with observed action [32]. However, the impact of observation of manipulation on the neural representation of tool-like objects is still unclear and is addressed in the present study.

In the present study we hypothesized that indirect object related experience, similar to active experience, would influence the perception of object affordances and induce tool representations in a left fronto-parietal network related to tool-oriented actions. To this end, we investigated the impact of observed manipulation on the processing of previously unfamiliar, manipulable objects [27], [29]. In three training sessions, participants observed one set of objects being actively manipulated by the experimenter (observation training objects, OTO), whereas a second set was visually explored (visually trained objects, VTO; see [27]). A third object set served as a control condition (not trained objects, NTO). Processing of pictures of the manipulable objects was assessed by means of functional magnetic resonance imaging (fMRI). To control for potential affordance-related activations elicited by the objects pre training, processing of object pictures was compared before and after training. We hypothesized that regions involved in observation of hand-object interactions [33] and affordance processing [8] would be more strongly activated by OTO than by VTO pictures after training. After training, we found a specific training-related brain activation increase for OTO in left iFG pars opercularis/triangularis and parietal supramarginal/angular gyrus.

## Materials and Methods

### Ethics statement

Participants were informed about the testing procedure and gave written informed consent. The study complies with the Declaration of Helsinki and was approved by the ethics committee of the Medical Faculty at the Ruhr University Bochum, Germany.

### Participants

19 healthy, right-handed students with a mean age of 23.21 years (SD = 3.36; range  = 18–31) participated in the study (11 females). All participants had normal or corrected-to-normal vision.

### Stimuli and experimental design

#### Object stimuli

Similar to the studies by [29] and [27], novel manipulable objects were used, constructed with K'nex (TM), a children's construction toy. Each object served one of six functions (“transport”, “destroy”, “push”, “pull”, “move” or “separate”) that could be performed on other small everyday objects (e.g. plastic cups, tea boxes, or table tennis balls). All novel objects were photographed from four different perspectives for the visual matching task (see below). A separate group of volunteers (N = 33) rated the object pictures in terms of similarity to real objects, visual complexity, and singularity, that is, how outstanding each object was compared to the other objects. Based on these ratings, the total group of objects was divided into three matched sets of objects, each comprising 12 different objects, two of each function.

#### Training

**Figure 1:**
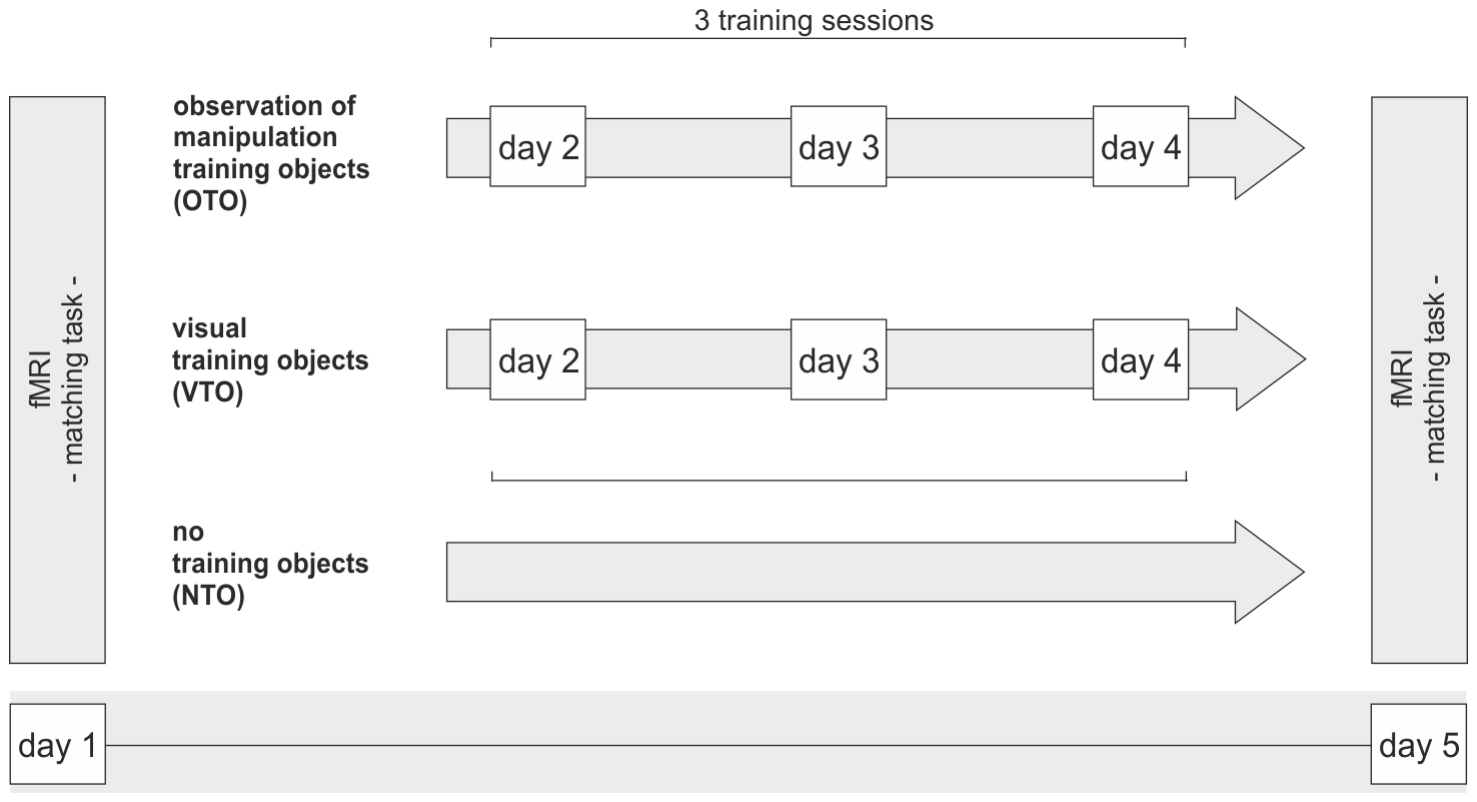
Experimental procedure.

Object-related training, which served to familiarize participants with the objects and to allow the formation of object representations, was divided into three training sessions of about 80 to 90 minutes that took place on three different days. On each training day, each participant received two qualitatively different types of object training with two different object sets during the course of one training session: “observation of manipulation training” with the first set of objects and “visual exploration training” with the second set (see Figure 1). The third, untrained object set served as a control and was only part of the visual matching task (see below). The allocation of object sets to “observation of manipulation training”, “visual exploration training” and “no training” was counterbalanced and randomized across subjects.

In “observation of manipulation training”, each object was first presented on a table in front of the participant before the concrete steps of manipulation were shown. The function of the object was named and subsequently the manipulation was demonstrated manually by the experimenter, simultaneously with a standardized verbal description of the discrete steps of manipulation. After demonstrating the use of the presented object, the participant was instructed to accurately observe the experimenter manipulating the object in the next 90 seconds and to furthermore count how often the experimenter performed the object-related action in this time period to ensure that the participant was paying attention.

In “visual exploration training”, the object was also placed on a table in front of the participant and the function was named. However, neither a description of the discrete steps of manipulation was provided nor was the manipulation shown. Instead, participants were asked to verbally describe the visual form of the object for 90 seconds and to pay attention to its visual structure, with no reference to its function or ways of possible manipulation. If a participant took less than 90 seconds for the description, the experimenter prompted the visual exploration asking specific questions about the constituents of the object (e.g. “How many blue bars does the object have?”), ensuring that the procedures for observation of manipulation and visual exploration were comparable in terms of the time spent with each object. In both training conditions participants were not allowed to touch or grasp the object.

### The visual matching task

**Figure 2:**
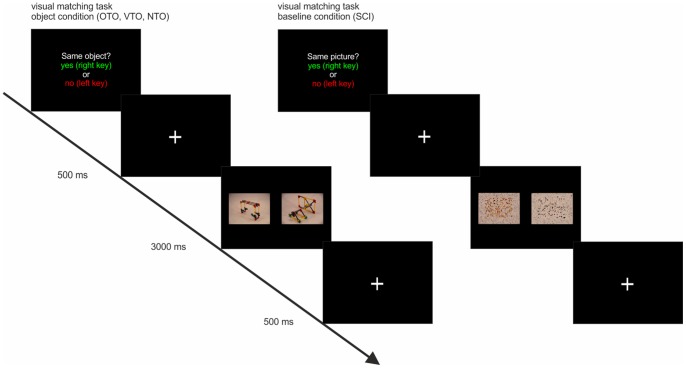
Example trials for object and scrambled image matching.

To examine training-induced changes in the neural correlates of processing pictures of the novel objects, the participants underwent fMRI pre and post training with an identical visual matching task, similar to the tasks used in previous studies [27], [29]. On each trial of the task, a fixation cross was first presented for 500 ms on a computer screen, which was projected on goggles worn by the subjects. Then a pair of pictures of the novel objects or a pair of scrambled images (SCI) of the objects, which served as a baseline condition, were shown for 3000 ms. Participants had to indicate whether the picture displayed the same object or not or, in case of the baseline condition, whether the SCI were identical or not. To respond, participants pressed either the index (for “identical”) or middle finger (for “not identical”) of the left hand during both tasks. Maximum response time was 3000 ms (see Figure 2). For the SCI, one picture per object was fragmented into 18 * 18 mosaic pieces which were then rearranged to yield a scrambled image of the object. To guarantee a comparable color distribution and distribution of the relevant information between center and periphery between SCI and unscrambled object pictures, the position of the 15×15 central mosaics was scrambled independently from the position of the peripheral mosaics.

The experiment was organized in a block design with four scan sessions, each comprising 16 blocks. Of the 16 blocks per session, four blocks contained trials exclusively showing OTO, VTO, NTO or SCI, respectively, with each block containing six trials. One block lasted 27.7 seconds. Half of the trials per block were matches showing the same object or the same SCI on both pictures and half were non-matches. Importantly, the pictures presented during a particular trial showed objects from different perspectives. For each object there were four different perspectives. During the experiment, each individual object was shown 16 times. Thus, each individual picture showing an object from a specific perspective appeared four times. Each SCI was presented five or six times. Stimulus timing, response and scanner pulse recording were controlled with Presentation software (Neurobehavioral Systems, Inc., Albany, California, USA).

After the second fMRI acquisition, subjects were successively shown pictures of all 36 objects outside of the scanner and participants were asked to specify by means of a printed questionnaire if the object had been part of the training or not. If yes, they were asked to indicate whether they had observed the manipulation of the object or visually explored the object (assignment of objects to the training condition). The interval between the last training session and the second fMRI acquisition was on average 3.21 days (SD = 2.90) and the mean time for accomplishment of the whole experiment including all five sessions was 11.26 days (SD = 5.36).

### Behavioral data analysis

Mean accuracy and reaction times in the matching task were analyzed with a repeated-measures ANOVA (Analysis of Variance) with the factors TIME (pre, post) and OBJECT SET (NTO, VTO, OTO). Correct assignment of object to training type after the second fMRI acquisition was analyzed by means of repeated-measures ANOVA with the factor OBJECT SET (NTO, VTO, OTO). In case of significant violation of sphericity, Greenhouse-Geisser corrected results and degrees of freedom are reported [34].

### Imaging parameters and analysis

Participants were scanned with a 3 Tesla Philips Achieva Scanner equipped with a 32-channel head coil. A high-resolution, three-dimensional anatomical T1-weighted MR image was acquired with a spoiled-gradient-recalled sequence during the first fMRI acquisition (pre training) with 220 slices, slice thickness 1 mm, TE = 3.74 ms, TR = 8.19 ms, flip angle  = 8° and an in-plane resolution of 1×1 mm. The T2*-weighted MR images during the functional sessions were acquired parallel to the anterior-commissure-posterior-commissural plane using an echo-planar (EPI) pulse sequence with 30 ascending slices of 4 mm thickness, TR = 2000 ms, TE = 30 ms, flip angle  = 90°, field of view of 224×240×120 mm and 2×2 mm pixel size. Each of the four functional imaging sequences started with 6 dummy scans that did not enter data analysis, and comprised 230 sequential volumes in total.

For preprocessing and statistical analysis, SPM8 (Statistical Parametric Mapping, Wellcome Department of Imaging Neuroscience, London, UK) was used in Matlab (Mathworks, Natick, Massachusetts, USA). Before preprocessing, all images were manually reoriented to the anterior commissure. Images were then corrected for slice timing, were realigned and unwarped [35], coregistered to the structural T1-weighted image and segmented and normalized to the Montreal Neurological Institute (MNI) standard space. Finally, an 8 mm FWHM Gaussian smoothing kernel was applied.

### General linear model

Data were temporally filtered with a non-linear high-pass filter with a 128 s cutoff. A global normalization was not performed.

#### First-level GLM

FMRI responses of each subject were modeled with a canonical hemodynamic response function, aligned to the onsets of blocks of trials belonging to one experimental condition. In a 4×2 factorial design with the factors TIME (pre, post) and CONDITION (NTO, VTO, OTO, SCI), t-Student contrasts were specified, each contrasting the different object sets (NTO, VTO, OTO) with the SCI baseline, separately for the pre and post training assessment. This procedure yielded six contrasts (NTO pre and post, VTO pre and post, OTO pre and post) for each subject, representing the activation for the respective object set relative to the baseline condition pre and post training.

#### Second-level GLM

On the second level, a full factorial design was specified with the within-subjects factors TIME (pre, post) and OBJECT SET (NTO, VTO, OTO), based on the first-level contrast images of the object sets relative to SCI baseline for each subject. Dependency and equal variance were determined for the factors. To control for between-condition differences in object memory, as reflected in the performance differences in assigning object photographs to the correct training condition after the second fMRI acquisition, (see Results section for details), a masking procedure was used on the second level. More specifically, individual subjects' performance scores for OTO, VTO and NTO, reflecting a measure of post training object familiarity, were entered as a regressor in a separate second level analysis comprising only the post training contrast images (see above). The brain activation pattern correlating with object familiarity was then used as an explicit mask for the second level analysis on object training effects to make sure that these effects were not related to object familiarity. Note that data of three participants were missing. For those, the mean scores of the remaining subjects were entered.

In our analysis, we aimed to identify brain regions that showed activation differences in response to pictures of specific object sets after, but not before training. We thus expected a specific activation increase for those objects the participants had experience with during training and were not interested in general activation increases across all conditions from pre to post training. Thus, activations of interest had to fulfill two criteria. First, the interaction contrast of the factors OBJECT SET and TIME had to be significant. Second, within the brain regions showing a significant interaction there had to be significant post training activation differences between object sets, examined by means of T-contrasts (e.g. OTO post >NTO post). At the same time, the respective contrasts between object sets before training were expected to not yield significant activations.

Statistics referring to whole brain analyses are reported. To keep type I (false alarms) and at the same time type II (missing true results) errors low during multiple comparisons, Monte Carlo simulation [36] was used to correct whole brain analyses for a threshold of p<.05, corrected. The threshold for single voxels was set at p<.001 and Monte Carlo simulation with 10.000 iterations was run, resulting in an extent threshold of 22 resampled voxels, defining a volume of 176 mm^3^. The SPM Anatomy Toolbox Version 1.8 was used to localize the activation peaks in MNI space [37].

As a further confirmation of the results, we applied small volume correction (SVC; [38]) both for the interaction F-contrast and the post training T-contrasts, focusing on relevant regions of interest. As already stated in the introduction, activation of a fronto-parietal network consisting of iFG (pars opercularis and triangularis) and iPL was expected for OTO stimuli. We hypothesized that regions involved in action observation of meaningful gestures, particularly in the left iFG [33], and potentially regions playing a role in affordance processing in the left parietal cortex [8] would be activated by OTO pictures after but not before training, indicating that action observation specifically modulated object processing in these areas. Hence, peak coordinates for SVC in the left inferior frontal cortex were derived from [33] (pars opercularis: x = −38, y = 8, z = 18; pars triangularis: x = −48, y = 36, z = 12). With respect to left iPL, peak coordinates from [8] were used (x = −54, y = −46, z = 30). Coordinates from Talairach space were transformed into MNI space (http://imaging.mrc-cbu.cam.ac.uk/downloads/MNI2tal/mni2tal.m) before applying SVC. An 8 mm sphere around the mentioned peak activations was used. For SVC, results with a corrected statistical threshold of p<.05 (FWE-corrected for the ROI in question) are reported.

## Results

### Behavioral Data

#### Performance in the matching task

**Figure 3:**
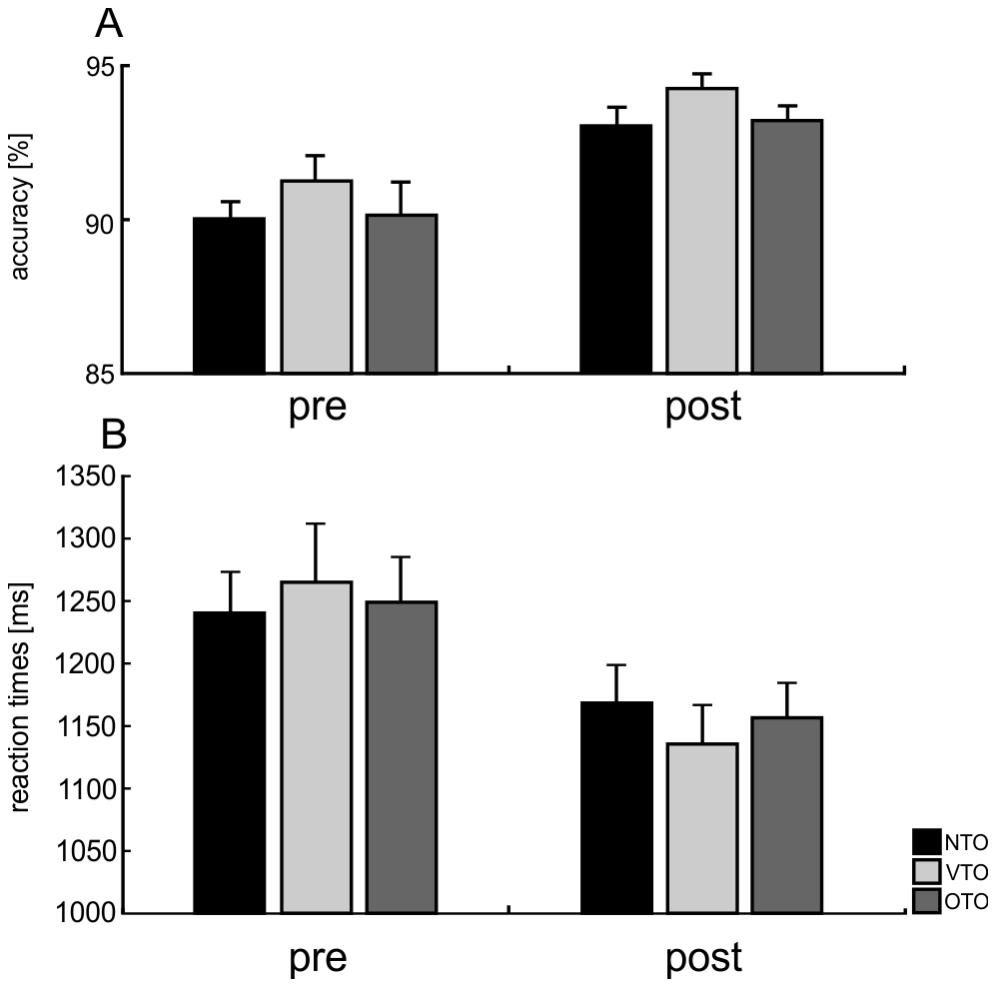
Mean accuracy and reaction times in the visual matching tasks before and after training. Error bars indicate standard error of mean.

Figure 3 displays mean accuracy and mean reaction times in the visual matching task before and after training. Behavioral data of two participants were excluded as a result of technical problems during response recording. For accuracy data, ANOVA with the factors TIME (pre, post) and OBJECT SET (NTO, VTO, OTO) revealed a main effect of TIME, indicating that participants responded more accurately after training than before training (F(1,16)  = 16.188; p = .001). No significant main effect of OBJECT SET and no significant interaction between the factors were found (all p>.179).

Analysis of reaction time data again revealed a significant main effect of TIME that indicated decreased response time post compared to pre training (F(1,16)  = 6.732; p = .020). The main effect of OBJECT SET and the interaction did not reach significance (all p>.232).

#### Assignment of objects to the training condition

After the second fMRI session, participants were asked to assign photographs of all objects to the training conditions (NTO, VTO, OTO; questionnaires of two participants are missing; a further questionnaire by one subject was not considered due to incompleteness). On average, participants correctly assigned 11.00 objects to NTO (SD = 1.00), 10.88 to VTO (SD = 1.71) and 9.50 to OTO (SD = 1.79). A one-factorial ANOVA revealed a significant effect of OBJECT SET (F(2,30)  = 7.26; p = .003) and post-hoc t-tests showed that the number of correct assignments of OTO was significantly reduced in comparison to VTO (t(15)  =  2.961; p = .010) and NTO (t(15)  = −4.240; p = .001), whereas no significant difference was found comparing VTO and NTO assignments (p = .699).

### Imaging data

As outlined in the Methods section, we identified brain regions on whole brain level for which brain activation during the visual matching task showed a) a significant interaction between the factors TIME and OBJECT SET and b) for which activation differences between object sets were seen after training, but not before training. Table 1 lists the brain activations fulfilling both these criteria at a corrected statistical significance threshold of p<.05 based on an extent threshold of 22 or more voxels defined by Monte Carlo simulations (see “Materials and Methods”).

**Table 1:** Brain regions showing activation differences between object sets after training and an interaction between the factors TIME and OBJECT SET.

		Peak coordinates MNI (mm)				
Brain region	Cluster size	x	y	z	Peak Z-Score	Uncorrected P-value (peak-level)
OTO>NTO						
L inferior frontal gyrus (pars triangularis)	196	−48	36	8	4.78	<.001
		−46	36	−2	4.16	<.001
		−42	28	8	4.14	<.001
L inferior frontal gyrus (pars opercularis)	159	−42	12	16	4.68	<.001
		−54	14	22	4.58	<.001
L rolandic operculum		−48	4	16	4.48	<.001
L angular gyrus	44	−48	−50	32	4.40	<.001
L supramarginal gyrus		−56	−52	30	3.93	<.001
OTO > VTO						
L precentral gyrus	116	−50	4	18	4.50	<.001
		−40	4	20	3.83	<.001
L inferior frontal gyrus (pars triangularis)	25	−44	38	2	3.73	<.001
VTO>OTO						
R precuneus	101	18	−42	8	4.58	<.001
R calcarine gyrus		22	−50	12	3.20	.001
L posterior cingulate cortex	95	−16	−46	8	4.27	<.001
L calcarine gyrus	30	−16	−60	18	3.57	<.001
L hippocampus		−20	−38	6	3.45	<.001

Please note that only clusters surviving the extent threshold of 22 voxels are reported, as revealed by Monte Carlo simulation to correct for multiple comparisons at p<.05. L =  left hemisphere, R  =  right hemisphere.

**Figure 4:**
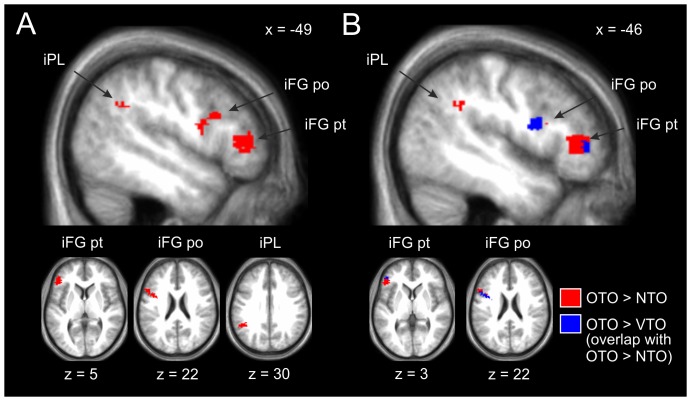
Significant post training activations projected on the mean T1 images of all study participants. Contrast A) displays OTO>NTO and B) displays OTO>VTO, overlapping completely with OTO>NTO (both p<.001, uncorrected). Note that all activations also had to show a significant interaction between the factors TIME and OBJECT SET. iFG = inferior frontal gyrus, pt  =  pars triangularis, po  =  pars opercularis, iPL  =  inferior parietal lobe.

Brain regions with stronger post training activation for OTO relative to NTO were found in a left-lateralized fronto-parietal network consisting of two clusters in iFG (pars triangularis and pars opercularis, extending into rolandic operculum) and one cluster in the angular gyrus/supra marginal gyrus (see Figure 4 A).

Significant activations also emerged for the contrast OTO>VTO. Parts of the iFG (pars triangularis) cluster which was active for the contrast OTO>NTO were found to be active also in this contrast with comparable peak-coordinates, but reduced cluster size (see Table 1). Furthermore, an activation peak was found in precentral gyrus, with the cluster again overlapping in part the cluster found for OTO>NTO (see Figure 4 B).

To identify brain regions that were specifically activated by visual exploration training, VTO-related brain activation was contrasted with NTO- and OTO-related brain activation. Relative to OTO, VTO elicited higher activation in a large right hemispheric cluster extending from the precuneus to the calcarine gyrus and in a left hemispheric cluster including posterior cingulate cortex (extending into hippocampus) and calcarine gyrus (see Table 1). No significant activation was found for VTO in contrast to NTO. Moreover, no activations for NTO relative to VTO or OTO were found.

To confirm the specific involvement of a fronto-parietal network of brain regions by OTO after training, SVC was applied at brain regions hypothesized to be involved in action observation and action selection for interactions between hands and objects, as already pointed out in the methods section. SVC was applied for the interaction contrast as well as for the contrasts between OTO on the one hand and VTO and NTO on the other hand after training. Both for the interaction contrast (pars triangularis, x = −50, y = 36, z = 8; 63 voxels; p = .002; pars opercularis, x = −40, y = 4, z = 18; 54 voxels; p = .003) and for OTO>NTO after training, activation within the left iFG was confirmed by SVC (pars triangularis: x = −48, y = 36, z = 8; 130 voxels; p<.001; pars opercularis: x = −42, y = 12, z = 16; 86 voxels; p<.001). Furthermore, we used coordinates from Grezes and Decety (2002) from an activation peak in parietal cortex. Again, a cluster of significant activation was found within this ROI for the interaction contrast (x = −48, y = −48, z = 30; 19 voxels; p = .018) and for OTO>NTO (x = −48, y = −50, z = 32; 159 voxels; p<.001).

Significant activations also emerged for the contrast OTO>VTO. SVC yielded significantly larger activations for OTO than VTO in the left inferior frontal cortex (pars triangularis: x = −48, y = 40, z = 6; 6 voxels; p = .012; pars opercularis: x = −44, y = 4, z = 18; 42 voxels; p = .005).

## Discussion

The present study aimed to elucidate the impact of observed manipulation on the neural processing of previously unfamiliar tool-like objects. In three training sessions, participants observed one set of novel objects being manipulated by an experimenter and visually explored a second set of novel objects to induce qualitatively different histories of object-related experience (observation training objects and visually trained objects – OTO and VTO, respectively). A third (control) set of novel objects was not part of the training (not trained objects - NTO). Before and after training, participants accomplished a visual matching task comprising pictures of the objects during which brain activity was assessed by means of fMRI. In accordance with our expectations, training effects on neural activity were seen in a left fronto-parietal network of brain regions. More specifically, the processing of OTO in contrast to NTO after training significantly activated left-hemispheric regions comprising parts of the left iFG (pars triangularis and pars opercularis) and the angular/supramarginal gyrus within the left iPL. Activations in left iFG occurred as well, albeit with smaller cluster sizes, for OTO in contrast to VTO, whereas no activation in the left iPL was detected for this contrast. Moreover, for VTO, compared to OTO, stronger activity within a bilateral cluster in precuneus and calcarine gyrus was found. Additionally, VTO in contrast to OTO activated left posterior cingulate cortex, extending into left hippocampus. Finally, no specific recruitment of brain areas was found for VTO in contrast to NTO or for NTO relative to the other object sets after training.

To our knowledge, the present study is the first to examine neural correlates of the effect of observed manipulation on the processing of novel tools. Two previous fMRI studies have shown that direct experience with previously unfamiliar objects via active manipulation leads to a stronger recruitment of fronto-parietal brain regions during the processing of pictures of the objects [27], [29]. This finding has been interpreted as support for sensory-motor theories of semantic object representations, proposing that conceptual knowledge is stored in or near brain regions associated with knowledge acquisition [21], [39]. The sight of objects would thus reactivate brain regions that were involved in active object manipulation. Theories of embodied cognition, the broader framework in which sensory-motor theories of conceptual knowledge formation are proposed, suggest that object concepts in semantic memory are represented within modal systems of the brain-related to perception and action. Within this framework, however, direct manipulation experience is not considered to be necessary for representations in motor regions to emerge (for review, see [24]). Similarly, models of praxis processing describe different access routes to action semantics, which provide a processing advantage when subjects are confronted with action-associated stimuli [18], [19]. In this respect, the current study adds further evidence in favor of embodied cognition theories, showing that also indirect object experience can alter object processing, possibly, in part, due to the induction of new object representations in the fronto-parietal action system.

Considering the induced activations in detail, the strongest training effects were seen in the iFG. In general, ventral premotor cortex, including pars opercularis and precentral gyrus, has been suggested to represent a human homologue of monkey area F5, containing, among others, so-called canonical neurons that discharge in the presence of graspable objects [40] and visuo-motor neurons which fire when a monkey sees a goal-directed action of another individual acting with an object (also referred to as “mirror neurons”; [41]–[43]). Activation of iFG pars opercularis was reported by studies investigating the processing of tool stimuli [3], [27] and when access to action concepts was required [44]–[46]. Furthermore, activation of pars opercularis and premotor cortex was reported in neuroimaging studies investigating neural correlates of action observation [47], [48], with left-hemispheric activation of ventrolateral premotor cortex in tasks requiring access to object representations and right-hemispheric recruitment during tasks requiring analysis of movements [47]. Recruitment of iFG pars opercularis has also been interpreted to reflect the extraction of action goals during grasp observation [33]. The inferior frontal activations observed for OTO in the present study might thus, at first view, represent canonical neuron firing, since the OTO were “transformed” from meaningless objects into graspable tools via training. The pattern of activation does, however, support a different view: When objects had only been visually explored, no recruitment of pars opercularis was evident after training. Hence, pars opercularis is not activated by all kinds of graspable objects alike. Consequently, the stronger post training iFG activations for OTO might be interpreted as a re-enactment of regions relevant for action observation, possibly related to previous action goal coding associated with the object.

A similar explanation may hold for the activation within pars triangularis for OTO. IFG pars triangularis activation was reported in studies on perception and discrimination of objects [4], [8] as well as during grasp observation [33], [48], [49]. With respect to the latter, Grafton et al. (1996) found activation at remarkably similar coordinates as the current study. Grasping as part of the observed object manipulation may thus have elicited activation in this region during the training procedure. After training, the object itself may then have been capable of eliciting an activation in or near this region.

It must be underlined that, both in the case of training studies involving active manipulation and of the present observational learning study, an interpretation in terms of a re-activation of brain regions that were active during knowledge acquisition while processing pictures of tool stimuli remains speculative, because brain activity during training was not assessed. Future studies will have to provide information on the degree of overlap of brain activations during acquisition of tool-related knowledge and tool processing. Another interesting topic for future research is whether the type of activation induced depends on the task subjects have to perform during active or observed manipulation.

Besides activation of frontal brain regions, parietal activation for OTO relative to NTO was found in the angular and supramarginal gyri (BA 40). Some previous studies investigating processing of tool-like stimuli reported activation of the parietal lobule [8], [27], [29]. Using transcranial magnetic stimulation, Tunik et al. (2008) showed the importance of the left supramarginal gyrus within the parietal lobule in goal-oriented object-directed action, and proposed that it plays a crucial role in the construction of action plans and in action selection for purposeful hand-object interactions. Corroborating this interpretation, Grafton et al. (1996), investigating neural correlates of grasp imagination, reported activation in BA 40 close to the activation seen in the current study, suggesting that the role of BA40 in action plan construction is independent of actual grasp execution. In further support of this view, Culham et al. (2006) proposed that the activation of the parietal cortex during tool observation is related to the strong affordances of tools for the various hand actions with the objects.

Patient studies also suggest that the parietal lobe plays an important role in tool use and manipulation [50], [51]. Randerath et al. (2010), for example, reported that patients suffering from apraxia characterized by deficits in tool use show a large lesion overlap in left supramarginal gyrus, whereas in patients mostly showing grasping errors the maximal overlap was seen in left iFG and angular gyrus. The authors propose that the angular gyrus provides information for grasping, while the supramarginal gyrus serves the integration of online and stored tool-related action knowledge. While this interpretation would be in line with the assumption that the parietal cortex stores tool representations along with information on manipulation, there are also theories claiming that the parietal lobe contribution is mostly inferring possible object uses on the basis the visual and tactile object properties affording certain actions (mechanical knowledge: [52]–[54]). An interesting double dissociation between object identification and object in patients suffering from semantic dementia or corticobasal degeneration does not support the notion of a separate action semantic system in the dorsal stream. Tool use in semantic dementia patients was characterized by a mechanical problem solving approach rather than by intact knowledge about proper tool use, probably guided by the intact parietal cortex. The corticobasal degeneration patient, with a prominent parietal involvement, on the other hand, showed chance level performance on mechanical problem solving. This observation suggests that viewing objects triggers a mechanical knowledge system, possibly located in parietal cortex, which extracts affordance cues to infer potential object use (e.g. [52]). With respect to the results of the present study, these findings might mean that the parietal activations do not reflect the induction of new semantic tool representations. Instead, a more general affordance-related process may be the trigger of this activation, in line with the finding that no activation of parietal cortex is found in the contrast of OTO compared to VTO. The latter result may be due to the fact that affordance-related activations were also triggered by VTO to some extent. At the same time, the parietal activations were clearly experience-dependent, as they occurred only after training and in contrast to the NTO. Thus, both observed manipulation and, to a lesser extent, visual exploration led to a strengthening of affordance-related processes during visual processing of tool-like stimuli. Learning about object function, which was provided to the subjects also in visual exploration training, may have played a key role for this process. Roy & Square (1985) and also Rothi, Ochipa & Heilman (1991, 1997) proposed that the action and the linguistic system are interconnected and that information about tool function as part of the conceptual praxis system can be acquired in different ways.

An alternative view on the role of the parietal cortex in motor performance has been put forward by Goldenberg (2009) in a recent review on the typical deficits associated with parietal brain damage in apraxia. Based on the observation that parietal lesions affect the imitation of meaningless gestures and the actual use of tools, but not the pantomime of tool use, he concludes that the parietal cortex does not store mental representations of movements. Rather, the degree of parietal recruitment depends on the “demands on categorical apprehension of spatial relationships between multiple objects” (p.1455), with the latter referring to objects, parts of objects or body parts [55]. This view was corroborated by the observation that parietal lesions affect the use of novel tools more than the use of common tools [14]. It is conceivable that increased object familiarity and/or the acquisition of object-function or object-action associations in the present study led to higher parietal involvement in the processing of object pictures, because an associated imagination of tool use required the apprehension of spatial relationships between the hands and the different object parts.

In the same study, Goldenberg and Spatt (2009) addressed also the differential role of the frontal and parietal cortex for apractic symptoms. Frontal lesions extending from the precentral gyrus to the middle and inferior frontal gyrus affected all functions assessed, from mechanical problem solving to functional knowledge and common tool selection and use. Accordingly, the authors concluded that the premotor cortex does not underlie the automatic planning of motor actions in response to the sight of tools, as suggested on the basis of imaging studies (e.g. [5]), but abstract aspects of movement planning [14].

The contrast VTO>OTO activated two large clusters in the medial parietal cortex, encompassing the calcarine gyrus bilaterally, in the left hemisphere protruding into the posterior hippocampus, and right precuneus. Additionally, VTO (in contrast to OTO) activated the left posterior cingulate cortex. Precuneus and hippocampus have been associated with episodic retrieval (e.g. [56], [57]). It cannot be excluded that VTO images elicited retrieval of training episodes, whereas OTO primarily activated non-episodic memory contents. This interpretation would also explain why participants showed better assignment of VTO than OTO pictures to the correct training condition. It must be underlined, however, that we accounted for this difference in performance in our fMRI analysis.

In summary, results from the current study show that a short history of observation of object manipulation can induce activation changes in the processing of previously unfamiliar tools in fronto-parietal brain regions associated with motor actions. These activations were elicited by the mere sight of the objects and may in part constitute a reactivation of regions active during the observation of manipulation itself, especially in the case of the iFG. Alternatively, the activations, especially those involving the parietal cortex, may be related to induced object affordances, possibly triggering the activation of a mechanical problem solving system, and/or a system processing spatial relationships. With the current study design we cannot distinguish which of these processes is responsible for the fronto-parietal activation changes following training, and future research is needed to clarify if these activation changes reflect the formation of new object representations or more general action-related processes.
